# Thoracoscopic lobectomy for pulmonary vein occlusion after radiofrequency catheter ablation of atrial fibrillation

**DOI:** 10.1186/s13019-016-0400-8

**Published:** 2016-01-19

**Authors:** Chien-Ming Lo, Hung-I Lu, Yen-Yu Chen, Jen-Ping Chang

**Affiliations:** Department of Thoracic and Cardiovascular Surgery, Kaohsiung Chang Gung Memorial Hospital, Chang Gung University, College of Medicine, 123 Tapei Rd, Niaosung District, Kaohsiung City, 833 Taiwan Republic of China

**Keywords:** Atrial fibrillation, Radiofrequency catheter ablation, Thoracoscopic lobectomy

## Abstract

**Background:**

Radiofrequency catheter ablation for paroxysmal atrial fibrillation is well established but not drawback free. Pulmonary vein stenosis is one of the complications and usually treated with stenting with the disadvantages of high re-stenosis rate and anticoagulant dependence.

**Case presentation:**

Herein, we present the case of a 47 year-old lady, who suffered from fever and hemoptysis due to right inferior pulmonary vein occlusion after radiofrequency catheter ablation for paroxysmal atrial fibrillation. Eventually, thoracoscopic right lower lung lobectomy was inevitable with satisfactory result.

**Conclusions:**

Pulmonary vein stenosis is a major complication after radiofrequency ablation of atrial fibrillation. High suspicion and early detection in patients with pulmonary manifestations are mandatory for salvage the injured lung in early. If delayed, surgical resection of the involved lung, especially through the thoracoscopic approach will eradicate the problem with minimal complication.

## Background

Radiofrequency catheter ablation is one of the recommended therapeutic options for the paroxysmal atrial fibrillation with low complication rate and promising success rate. However, pulmonary vein stenosis is one of the major complications of this procedure and could be fatal [1]. Hemoptysis, dyspnea, intractable cough, and recurrent pneumonia are the most common manifestations [2]. Radical procedure like lobectomy may be necessary in the case of medical treatment failure, recurrent pneumonia or sepsis [3, 4]. We reported on a 47 year-old lady with recurrent pulmonary events after radiofrequency catheter ablation of paroxysmal atrial fibrillation. The diagnosis of right inferior pulmonary vein occlusion was delayed and eventually, thoracoscopic right lower lung lobectomy was inevitable with satisfactory result.

## Case presentation

A 47 year-old barber lady with three-year history of paroxysmal atrial fibrillation was admitted on 2 June 2013 for recent incremental attacks of palpitation. Physical examination and laboratory tests were all unremarkable. On 4 June 2013, the electrophysiological study using EnSite^TM^ Velocity^TM^ (St. Jude Medical One St. Jude Medical Drive, St. Paul, MN 55117 USA) through the transseptal approach confirmed that the paroxysmal atrial fibrillation was originating from the right superior and inferior pulmonary veins. Subsequently, 20–30 Watts (45° Celsius for 30 to 40 s) of radiofrequency energy was applied to atrial locations of the four pulmonary veins utilizing a 7 F quadripolar irrigating ablation catheter. The successful ablation was confirmed by complete antegrade and retrograde electrophysiological study using incremental pacing and extrastimulus technique during isoproterenol administration. The patient was discharged six days later with uneventful course until 10 September 2013, a febrile episode associated with productive cough and mild dyspnea happened. Right lower lobe pneumonia was found by chest roentgenography and computed tomography. The patient was discharged in improved condition after antibiotic treatment for two weeks.

**Fig. 1:**
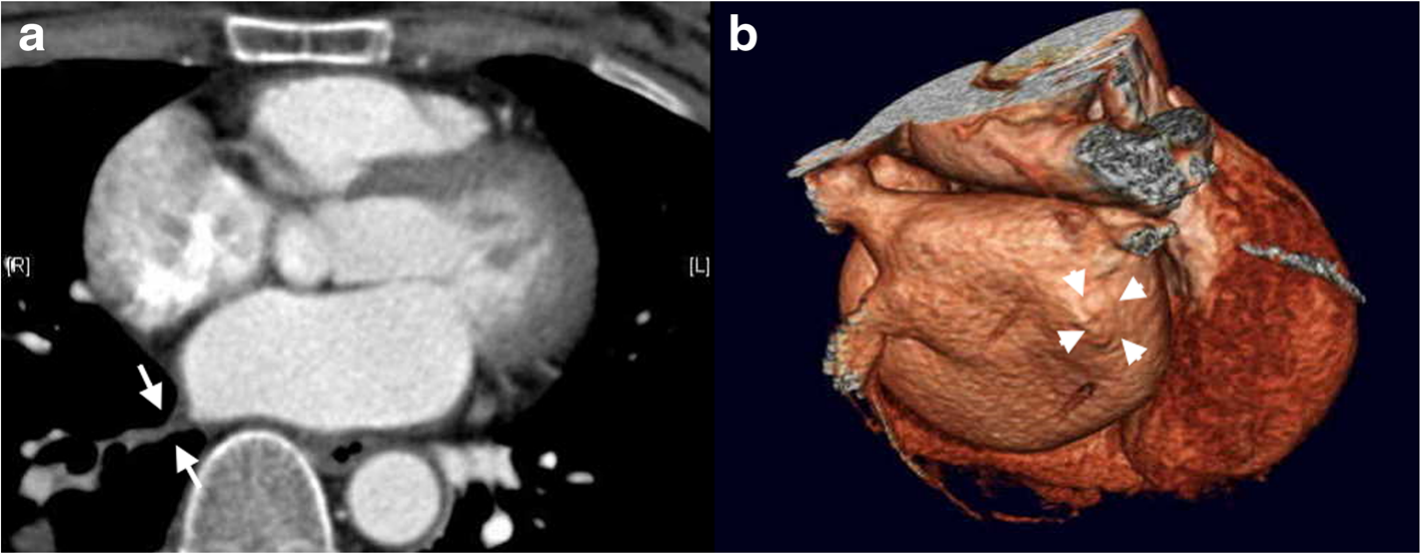
**a**: A cardiac computed tomographic angiogram revealed right inferior pulmonary vein occlusion (between arrows). **b**: A three-dimensional cardiac computed tomographic angiogram showed absent right inferior pulmonary vein (arrow heads)

Hemoptysis and chest discomfort occurred 23 November 2013. An emergent chest roentgenogram revealed right lower lung consolidation. The bronchoscopic study was unremarkable. A cardiac computed tomographic angiogram was performed on the presumption of iatrogenic pulmonary vein injury and revealed right inferior pulmonary vein occlusion on 28 November 2013 (Fig. 1a and b). Because of the totally occluded pulmonary vein, stenting was aborted due to high technical demand, high re-stenosis rate, and need of almost life-long anticoagulant therapy. On 10 December 2013, six months since the radiofrequency ablation of the paroxysmal atrial fibrillation, thoracoscopic right lower lobectomy was performed with four-port technique. Severe inflammatory adhesion of the right pulmonary hilar region and totally fibrotic right inferior pulmonary vein were noted. The postoperative course was uneventful and the patient was discharged on eleventh postoperative day. Chest roentgenogram was followed every three months and cardiac computed tomographic angiogram was followed every six months during the follow-up period. The patient has been free from both pulmonary and cardiac events 18 months postoperatively and 24 months after the radiofrequency ablation of the atrial fibrillation.

On the microscopic examination, extensive hemorrhagic necrosis with acute and chronic inflammatory cell infiltration, interstitial fibrosis, and vascular thrombosis were noted around the right inferior pulmonary vein.

## Discussion

Besides the medical rate control and surgical ablation, radiofrequency catheter ablation is another therapeutic option for the paroxysmal atrial fibrillation with intractable symptoms. The procedure related overall complication rate is 6.29 %. Among them, the cardiac complications include myocardial infarction, pericardial complications and various iatrogenic cardiac injuries had been reported. The pulmonary vein injury is one of the major adverse events of this procedure [1–3].

Pulmonary vein stenosis can be under-recognized because the manifestations are often obscure in the early stage or in cases with limited involvement. Repetitive or progressive deterioration of non-specific respiratory manifestations suggest that the iatrogenic pulmonary vein stenosis getting significant. The first 2 cases of pulmonary vein stenosis after radiofrequency ablation of atrial fibrillation were successfully treated with balloon dilation [5]. Since then, this complication is usually treated with percutaneous transluminal pulmonary vein balloon dilatation or stenting with the demerits of significant re-stenosis rate and need of long-term antithrombotic therapy [2]. In the limited cases with irreversible occlusion with significant symptoms, dilation and stenting can be not effective and eventually requiring pulmonary resection [4, 6, 7].

In our patient, because of the totally occluded pulmonary vein, the percutaneous transluminal approach was aborted and the surgical resection of the injured lung would be the final option. Certainly, this method would eradicate the lesion on the cost of sacrifice of pulmonary capacity. Otherwise, although technically demanding, surgical pulmonary venous angioplasty might be another salvage option. However, in the chronic case with severe inflammatory adhesion, this procedure might be impossible. Furthermore, the injury created by the radiofrequency energy was reported to result in irreversible venous and arterial medial thickening and intimal hyperplasia throughout the lung with consolidation that prohibited the lung sparing strategies [6].

## Conclusion

In conclusion, pulmonary vein stenosis is a major complication after radiofrequency ablation of atrial fibrillation. High suspicion and early detection in patients with pulmonary manifestations are mandatory for salvage the injured lung in early. If delayed, surgical resection of the involved lung, especially through the thoracoscopic approach will eradicate the problem with minimal complication.

## Informed consent

Written informed consent was obtained from the patient for publication of this Case report and any accompanying images. A copy of the written consent is available for review by the Editor-in-Chief of this journal. This case study was approved by Institutional Review Board for Kaohsiung Chang Gung Memorial Hospital (104-7429B).
